# Sinup is essential for the integrity of centrosomes and mitotic spindles in zebrafish embryos

**DOI:** 10.1080/19768354.2017.1308438

**Published:** 2017-04-13

**Authors:** Kyeong-Won Yoo, Sateesh Maddirevula, Ajeet Kumar, Hyunju Ro, Tae-Lin Huh, Myungchull Rhee

**Affiliations:** a Department of Biological Sciences, College of Biosciences and Biotechnology, Chungnam National University, Daejeon, South Korea; b Department of Genetics, King Faisal Specialist Hospital and Research Center, Riyadh, Saudi Arabia; c College of Natural Sciences, School of Life Sciences and Biotechnology, Kyungpook National University, Daegu, South Korea

**Keywords:** Sinup, phosphorylation, centrosomes, spindle fibers

## Abstract

Fish lineage-specific gene, *sinup* [Siaz-interacting nuclear protein], modulates neural plate formation in embryogenesis and shares homology with human TPX2 protein, a member of the vertebrate mitogen-activating protein family. In spite of the presence of the TPX2 domain in Sinup, its cellular function has been unknown. As an initial approach to this question, we expressed Sinup by injecting *sinup-EGFP* mRNAs into zebrafish embryos at the one- to two-cell stage. First of all, Sinup-EGFP was associated with centrosomes and mitotic spindles. In particular, Sinup was localized to the spindle poles and midbody microtubules during the period between anaphase and cytokinesis. Second, various deleted mutants of Sinup-EGFP failed to be associated with the centrosomes and mitotic spindles. Third, a Sinup mutant, where the 144th Serine residue was converted to alanine, not only disturbed the mitotic spindle organization, such as multipolar spindles, fragmented spindle poles, and flattened spindles, but also arrested the cell cycle at metaphase and cell movement. Finally, Sinup is phosphorylated by Aurora A and the 144th Serine mutant of Sinup is partially phosphorylated by Aurora A kinase. We thus propose that Sinup is an essential element for the integrity of centrosomes and mitotic spindle fibers as well as for the normal process of cell cycle and cellular movement in vertebrate embryos.

## Introduction

Vertebrate embryogenesis is a complex process employing various cellular signaling pathways and co-ordinations among cells. Particularly in the early cell cleavage to gastrulation period, cells derived from the three germ layers undergo rapid rearrangement and movement to shape the body axis and shapes (Keller et al. 1991). Total vertebrate body architecture, axis, and shape are determined by cellular activities such as cell division, microtubule cytoskeleton rearrangements, proliferation, cell movements, and cell intercalations; however, all these events are largely influenced by cellular environment and localization in vertebrates (Roszko et al. 2009). Morphogens, such as Wnt, Nodal, Bmp, FGF, and Notch, determine the cellular movement in embryogenesis (Ip & Gridley 2002; Myers et al. 2002; Schier & Talbot 2005; Krahn & Wodarz 2009). Microtubules and cytoskeleton individuate the cellular behavior, while centrosomes influence the shape and length of the spindle by stabilization and destabilization (Karsenti & Vernos 2001; Mitchison et al. 2005).

During gastrulation of zebrafish and *Xenopus,* cytoskeleton rearrangements and cell movement process are governed by the planar cell polarity (PCP) pathway, which is regulated by non-canonical Wnt signaling. Microtubules and cytoskeletal rearrangements are major cellular events in vertebrate embryogenesis. PCP pathway-related genes, such as Prickle, c-Jun N-teriminal Kinase, Van Goah-like protein 2, and Rho kinase in the nucleus, modulate microtubules and cytoskeletal rearrangement for proper cell shape and movement (Shulman et al. 1998; Park & Moon 2002; Marlow et al. 2002; Montero et al. 2005). A zebrafish mutant *trilobite,* defective in the homolog of Van Gogh-like protein 2, shows severe cell movement defects (Park & Moon 2002). Zebrafish *prickle* regulates the cell movement during embryogenesis (Veeman et al. 2003).

The Solnica-Krezel group recently reported that Wnt/PCP signaling controls the intracellular position of the microtubule organization center (MTOC) for gastrulation convergence and extension (CE) movements in zebrafish embryos. The centrosome position determines the axis of polarity and microtubules mold the polarization of the cells along the anterior and posterior axes (Sepich et al. 2011). Centrosome-associated proteins, such as Dynein, Lis1, Dynactic, Polo, and Aurora A kinase, are highly conserved and involved in similar functions in the cells (Knoblich 2008; Siller & Doe 2009). Aurora A kinase, a serine/threonine kinase, regulates the cell division by modulating the centrosome, cytoskeleton, and spindle polarity in association with spindle-associated cofactors, such as Ajuba, HBora, PAK1, and TPX2 (Barr & Gergely 2007; Eckerdt et al. 2008).

The ubiquitin proteasome system critically regulates various mechanisms such as cell polarity and neurogenesis (Anuppalle et al. 2013a, 2013b). Siah as E3 ligase modulates embryonic patterning (Kang et al. 2014) and its interacting partner Sinup is involved in the neural plate formation (Ro et al. 2005). Despite the neurogenic roles of Sinup, its cellular functions remain unclear. We are reporting the functional roles of Sinup in cell movement during gastrulation as well as in centrosome integrity, based upon studies on its cellular localization, morpholino-based knockdown, misforced expression, and phosphorylation assays.

## Materials and methods

### Animal care and maintenance

Wild-type zebrafish was obtained from the Korea Zebrafish Organogenesis Mutant Bank (ZOMB) and maintained under standard conditions as described in Westerfield (2000). Embryos were obtained by natural spawning, raised at 28.5°C, and staged as described in Kimmel et al. (1995).

### Cloning of *sinup* and generation of mutant constructs

*Sinup* was isolated from 30% epiboly cDNA with *sinup-*specific primers by using pfu turbo as described (Ro et al. 2005), and cloned into pGEM^®^-T Easy Vector Systems. DNA fragment encoding *sinup* was sub-cloned into the pCS2-EGFP vector. Deleted mutants and point mutation of *sinup* were generated with inverse PCR under standard condition with specific primers (Supplementary Information).

### Morpholino, mRNA synthesis, microinjection, and *in situ* hybridization

*Sinup-*specific translation-blocking antisense morpholino and five mismatched control morpholino oligonucleotides were obtained from GeneTools (Philomath, USA) as the sequences were described in Ro et al. (2005). *sinup-EGFP* mRNAs were synthesized with linearized pCS2–*sinup*-*EGFP* using mMESSAGE kit (Ambion) and injected into wild-type embryos at the one- to two-cell stage as described in Anuppalle et al. (2013a). For *in situ* hybridization, probes for *myoD* (Weinberg et al. 1996), *pax2.1* (Krauss et al. 1991), *gsc* (Stachel et al. 1993), *dlx3* (Akimenko et al. 1994), *hgg* (Thisse et al. 1994), and *ntl* (Schulte-Merker et al. 1994) were synthesized as described in Lee and Rhee (2009).

## Cell culture and transient transfections

COS-7 cells were cultured in Dulbecco’s modified Eagle medium (DMEM) supplemented with 10% heat-inactivated fetal bovine serum (FBS) (v/v) and with antibiotics. The cultured cells were grown in a 5% carbon dioxide humidified atmosphere at 37°C. pCS2+*sinup-EGFP* was transfected with Transfectin^™^ lipid reagent (Bio-Rad) and observed under a Zeiss confocal microscope used for fluorescent image acquisition.

## Kinase assay

Glutathione S-transferase (GST)-Sinup fusion protein was prepared as described in Ro et al. (2005) and *in vitro* kinase assay was performed as described in Jang et al. (2008).

## Confocal scanning

Sinup-EGFP-injected embryos were embedded in 1% low-melting agarose at the sphere stage and time-lapse analysis was performed with a Zeiss LSM-5 confocal microscope.

## Results

**Figure 1.**
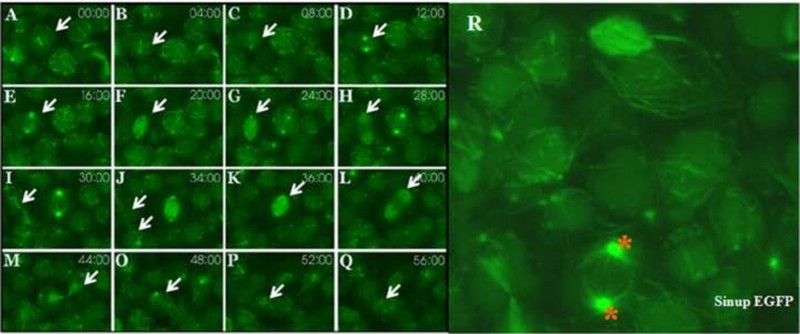
Sinup is localized in the centrosomes and mitotic spindles: *sinup-EGFP*-injected embryos were scanned with a confocal microscope with animal pole view. (A–Q) Sinup was localized in the mitotic spindle organization throughout the cell division. (R) Magnified expression domain of *sinup* transcripts. Asterisks indicate centrosome and arrow indicates microtubules.

Sinup was initially identified as a Siaz-interacting nuclear protein in the mammalian cell line because it is localized within the nucleus in the *sinup-EGFP*-transfected COS7 cells (Ro et al. 2005). To further investigate its cellular distribution *in vivo*, mRNAs *in vitro* transcribed from *sinup/EGFP* fusion construct were injected into zebrafish embryos at the one- to two-cell stage. Sinup-EGFP was co-localized with the centrosome and mitotic spindle throughout mitosis (Figure 1(R), (A–Q)). In particular, Sinup-EGFP was in the poles at prometaphase (Figure 1(A,B); arrows) and at interphase (Figure 1(C); arrow). Sinup-EGFP was present along the microtubules during early anaphase and anaphase (Figure 1(E–H); arrows), which last till cytokinesis (Figure 1(I); arrow). Sinup-EGFP continued to be present in the poles in daughter cells (Figure 1(J); arrow). It is thus clear that Sinup-EGFP is associated with the center of spindles, and midbody microtubules throughout the cell cycle.

**Figure 2.**
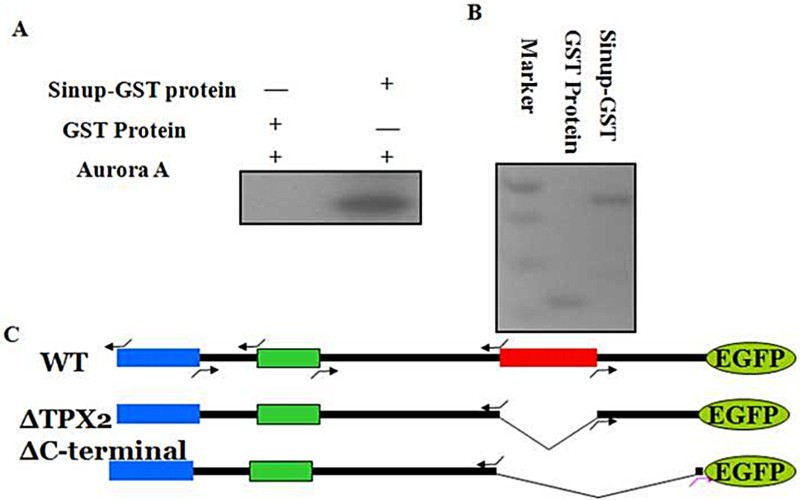
*In vitr*o kinase assay with Sinup recombinant protein: (A) Sinup-GST recombinant proteins were incubated with Aurora A kinase in the presence of γ− [P^32^] ATP for 30 min at 37°C and analyzed in 12% SDS-PAGE. Incorporation of P^32^ into Sinup was measured by autoradiography. (B) Purified recombinant proteins stained with Coomassie brilliant blue on SDS-PAGE gel. (C) Schematic drawing of the *sinup-EGFP* fusion protein construct and its derivatives (deletion constructs). There are several domains; bacterial Ig-like domain 1 (blue), bipartite nuclear localization signal (green), and Tpx2 homology motif (red).

Sinup is a nuclear protein with various domains, such as bacterial Ig-like domain-1, bipartite nuclear localization signal, and Tpx2 homology motif. For better understanding the biological roles of the various domains of Sinup in regard to its association with the centrosome and spindle fibers, we generated a series of Sinup deletion constructs fused with EGFP (Figure 2(C)). mRNAs *in vitro* transcribed from each of the deletion mutants were microinjected into embryos at the one- to two-cell stage to examine the biological effects of the mutants on the formation of intracellular organelles as well as cellular movement. However, SinupΔTPX2-eGFP (TPX2 domain deleted) and SinupΔC-eGFP (C-terminal region deleted) did not cause observable changes in the phenotypes of interest (data not shown). We then payed attention to a phosphorylation site at the 144th residue, serine within the TPX2 domain, because testis-specific protein (TPX) domains have been known to stabilize spindle association and integrity in human cells (Giubettini et al. 2011). In order to investigate the functions of Sinup via phosphorylation within the TPX domain, Sinup-GST recombinant proteins were generated and subjected to *in vitro* phosphorylation assay. Surprisingly, Sinup was effectively phosphorylated by Aurora A kinase in comparison with the control (Figure 2(A)).

**Figure 3.**
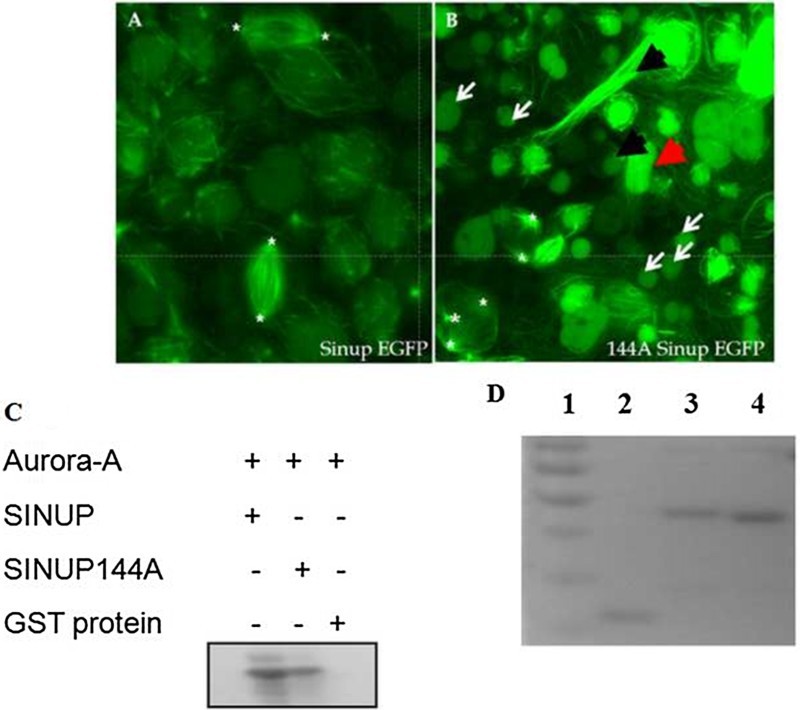
Effect of misforced expression of *sinup* on spindle organization. (A) *sinup*-*EGFP*-injected embryo. (B) *sinup144A-EGFP*-injected embryo. The embryos were scanned at the sphere stage with a confocal microscope. The embryos injected with *sinup144A-EGFP* showed disruption in the mitotic spindle organization, orientation, and polarity of the spindle pole. Multipolar spindles were labeled with white star. (C) *In vitro* phosphorylation assay of Sinup144A recombinant protein. Phosphorylation of Sinup144A by Aurora A was greatly reduced in comparison with that of wild-type Sinup. **(**D) Purified recombinant proteins were stained with Coomassie brilliant blue on SDS-PAGE gel. 1: Marker (Fermentas #SM0671), 2: GST protein, 3: Sinup-GST, 4: Sinup144A-GST.

We further tested the biological effect of the phosphorylation of the 144th residue of Sinup. We generated a *sinup* mutant fused with EGFP (Sinup144A-EGFP) where serine at the 144th position was replaced with alanine. mRNAs encoding Sinup144A-EGFP were microinjected into zebrafish embryos at one- or two-cell stages for its overexpression. Striking changes were observed in the spindle pole organization and polarity of spindle fibers (Figure 3(B)), such as multipolar spindles (Figure 3(B); asterisks), fragmented spindle poles, and flattened spindles (Figure 3(B); black arrow heads). Furthermore, cells failed to enter the metaphase in the Sinup144A-EGFP overexpressed embryos (Figure 3(B); white arrows). Taken together with the biochemical evidence for phosphorylation of serine at the 144th residue, it is conceivable that phosphorylation of Sinup at the144th residue is critical to the integrity of centrosomes and spindle fibers.

**Figure 4.**
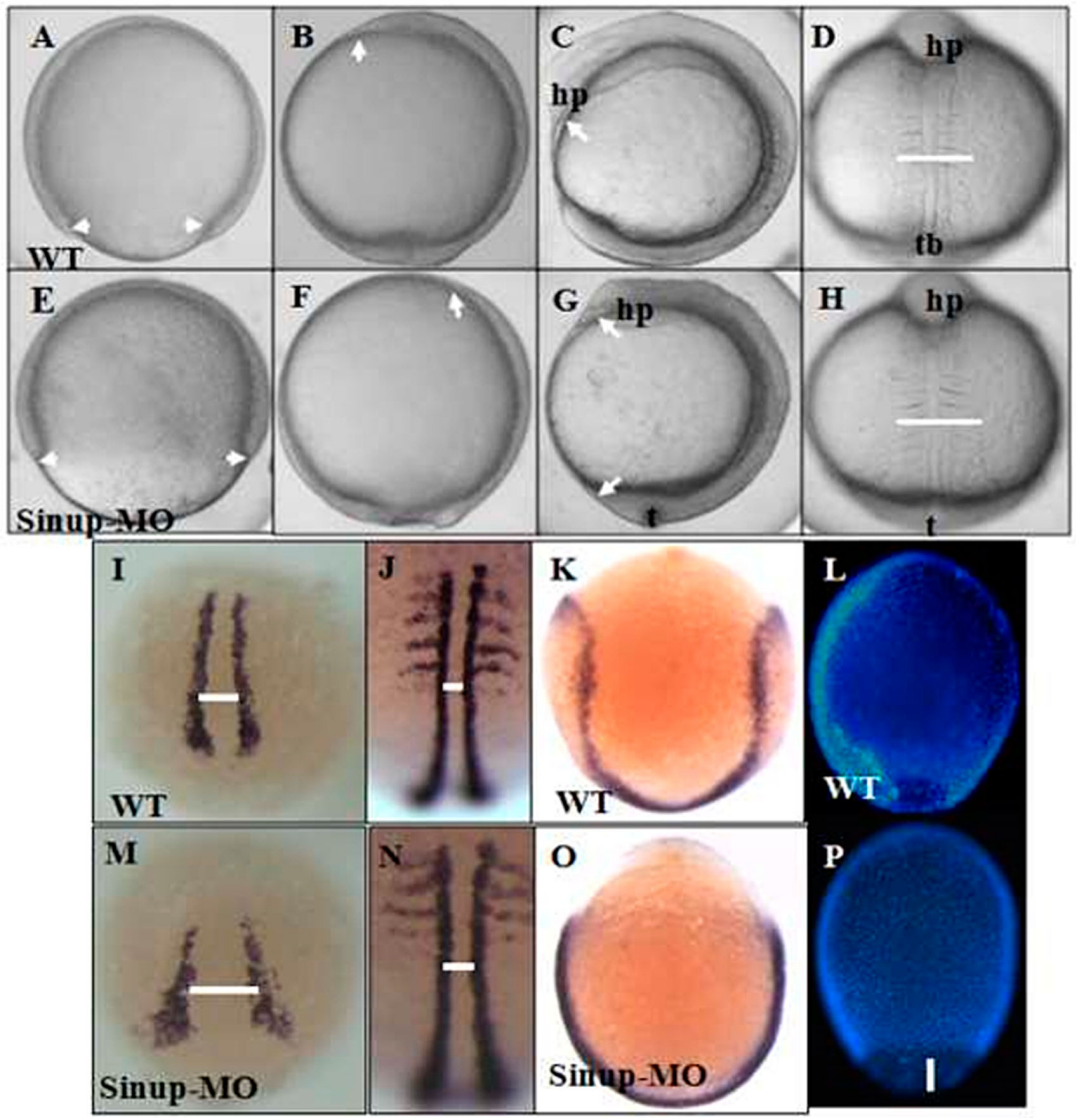
Knockdown of *sinup* expression with *sinup*-specific morpholino (*sinup* MO) alters cell movement along CE. *sinup* MO was injected in one- to two-cell stage embryos. Control embryos and the injected embryos were fixed at 90% epiboly (A,E), tail bud (B,F,I,K,L,M,O,P), and five-somite stages (C,D,G,H,J,N). The embryos were subjected to whole-mount in situ hybridiation with *myoD* (I,M), *pax2.1* and *myoD* (J,N), *dlx3* (K,O), and DAPI staining (L,I). (A–D) and (I–L) are wild type; (E–H) and (M–P) are *sinup* MO-injected embryos. Lateral view: A–C,E–G, L–P; dorsal view: D,H,I–K, M–O.

It has been reported that forced expression of *sinup* during early embryogenesis changed significant expression patterns of the marker genes (Ro et al. 2005). To elucidate biological roles of Sinup in early embryogenesis, *sinup*-specific antisense morpholino (*sinup* MO) was injected into embryos at the one- to two-cell stage for its translational inhibition. *sinup* MO blocks the synthesis of the Sinup-EGFP protein, while the mismatch *sinup*-specific morpholino (*sinup* 5 mm MO) (data not shown). While *sinup* MO displayed developmental defects in cell movements, the phenotypic defects appeared in the deep cells and enveloping layer cells of the embryos at the late gastrula stage (Figure 4(A,E)). *sinup* MO exhibited disposition in the prechordal plate, and the shorter anterior and posterior axis (AP) (Figure 4(B,C,F,G)) as well as enlarged mediolateral axis (ML) (Figure 4(D,H)).

In order to gain insight into the molecular elements underlying the phenotypic alteration, we analyzed expression patterns of molecular marker genes *myoD* and *distal-less homeobox 3* (*dlx3*) in *sinup* MO. The expression pattern of *myoD* in *sinup* MO found major changes in the CEs in the somites as well as in the paraxial mesoderm of early somites (Figure 4(I,J,M,N)). On the other hand, the expression domain of *dlx3*, a marker for neural plate boundary, became widened in the boundary (Figure 4K,O)). We thus further analyzed the movement of the enveloping layer cells as well as deep cells using 4',6-diamidino-2-phenylindole (DAPI) staining, and discovered that cellular movement was severely retarded in *sinup* MO (Figure 4(P)) compared to that in control (Figure 4(L)). Based upon these observations, we propose that Sinup is an essential component to the formation and integrity of centrosomes and microtubules for proper cellular movement in early embryogenesis.

## Discussion

Sinup is localized in the centrosomes and mitotic spindles along the cell cycle. The vertebrate body plan is constituted during gastrulation by cytoskeletal rearrangements, cell fate specification, morphogenetic movement, and cell movement, but all these are governed by the convergence extensions (Narasimha & Leptin 2000; Myers et al. 2002). In embryogenesis, a large set of genes tightly regulate cell division and thereby organogenesis. Cell cycle components are critical to cell fate determination throughout embryogenesis. Mitotic spindle organization and its associated protein kinases play key roles in such processes. In addition to this, bipolar microtubule structural organization is strictly required for chromosomal segregation during mitosis (Compton 2000). In vertebrate animals, centrosome position determines the fate of the cytoskeleton and AP axis and ML axis of cell movements during gastrulation, while AP axis cell movement is established by the microtubules (Compton 2000; Sepich et al. 2011). Interestingly, Sinup is localized with spindles and midbody microtubules during cytokinesis. Rearrangement of cytoskeleton regulates the cell division in late cytokinesis during cell division requires membrane delivery to the furrow (Glotzer 2003). Mitotic spindle-associated proteins/kinases regulate the cell movement to determine the AP and ML axes of vertebrate animals. In *Xenopus*, Wee2 kinase regulates the cell movement by phosphorylating Cdk (Leise & Mueller 2004). It is most probable that Sinup as a centrosome and mitotic spindle-associated protein plays pivotal roles in the integrity of centrosomes and mitotic spindles to regulate cell movement in early embryogenesis.

Sinup is phosphorylated at the 144th residue, serine by Aurora A kinase (Figure 2(A)). It is well known that TPX2 is associated with mitotic spindles and involved in Aurora A stabilization in *Xenopus* and *C. elegans* (Wittmann et al. 2000; Ozlu et al. 2005). Aurora A kinase as a mitotic spindle-abundant kinase plays an important role in the mitotic spindle integration and stability. Activation of Aurora A is the major event in regulating spindle stability and cell division, while TPX2 is a mandatory protein for the activation of Aurora A (Bayliss et al. 2003; Mitchison et al. 2005). Centrosomes or mitotic spindles are associated with several proteins which are involved in the cell cycle process, such as CHK2, TPX2, Astrin, and Aurora A kinase (Barr & Gergely 2007). Aurora A kinase regulates cell polarity, spindle elongation, spindle assembly, and centrosome maturation in *C. elegans* (Ozlu et al. 2005)*.* We not only demonstrated here that the 144th Serine residue of Sinup was partially phosphorylated by Aurora A kinase (Figure 3(C)), but also showed that misforced expression of Sinup144A caused severe alterations on mitotic spindles by disturbing the polarity of spindles, leading to multiple spindle organizations (Figure 3(B)). While it appears that phosphorylation of Sinup at the 144th residue governs the cellular movements during gastrulation, it is worthwhile to search for other phosphorylation sites within the TPX2 domain of Sinup.

CE movements are triggered by cellular signals from the margin of epiboly that drive the motor proteins during vertebrate gastrulation (Solnica-Krezel & Driever 1994 ; Solnica-Krezel 2006). During the early gastrulation of zebrafish tubulin cytoskeleton, actin and epiboly shape the embryo. As we witnessed that elimination of Sinup severely hindered the cell movements in the epiboly period (Figure 4(A–D)), Sinup might be a critical element for epiboly progression. Current reports show that various upstream signaling molecules, such as Wnt (Wnt11, Wnt5a), Ephirin, and Platelet-derived growth factor/PI3 k, regulate complex cytoskeletal arrangements and cellular movements during gastrulation, which involve a dynamic process of microtubule arrangement. It is thereby worthwhile to ask if Sinup could be a downstream target of the signaling pathways.
